# The effects of pseudophosphorylation on *Vitis riparia* dehydrin structure and function

**DOI:** 10.3389/fpls.2026.1946970

**Published:** 2026-10-08

**Authors:** Marijke R. Murray, Kelly F. Boddington, Steffen P. Graether

**Affiliations:** Department of Molecular and Cellular Biology, University of Guelph, Guelph, ON, Canada

**Keywords:** abiotic stress, dehydrins, function, phosphorylation, pseudophosphorylation, structure

## Abstract

Plants undergo a wide range of deleterious effects as a result of exposure to abiotic stress, such as cold, salinity, and drought conditions. Some of these effects include protein denaturation and aggregation, membrane leakage and phase transitions, and DNA damage. One protective measure includes the expression of dehydrins, which are members of the group 2 LEA protein family. Several studies have shown that the S-segment, a serine-rich tract found in many dehydrins, can be phosphorylated *in vivo*. The main functional role of this phosphorylation appears to recruit the dehydrin to the nucleus, though this is not an absolute requirement. Here, we investigate whether pseudophosphorylation (i.e. the substitution of serine residues with glutamate) had any effect on the protection of proteins, membranes and DNA using VrDHN1 as a model YSK_2_ dehydrin. The changes resulted in improved protection of lactate dehydrogenase from freeze-thaw damage and caused an increase in α-helicity after binding to SDS micelles. Pseudophosphorylation was found to reduce DNA binding but had no effect on the protection of DNA from ROS damage. In addition to a possible role in nuclear localization, phosphorylation may result in a tuning of dehydrin function toward improved protective ability.

## 1 Introduction

Abiotic stressors such as cold, salinity and drought can cause multiple types of damage to biomolecules in plants. All three of these stresses cause cellular dehydration, either directly through drought conditions, or indirectly through water efflux or intracellular ice formation, resulting in a lack of liquid water in the cell. This can lead to multiple deleterious effects, including a weakening of the hydrophobic effect, causing protein instability and denaturation (Steponkus, 1984). As well, increasing levels of reactive oxygen species (ROS) can surpass the antioxidizing capacity of the cell, damaging proteins, nucleic acids and membranes (Mittler, 2002), resulting in protein misfolding and denaturation, DNA base-pair mismatches and lipid peroxidation. Freezing stress can result in membrane phase transitions, an increase in permeability, and a loss of fluidity (Cruz De Carvalho, 2008).

Plants have evolved a wide range of mechanisms to protect against these negative effects. One component of this stress response is the expression of dehydrins (dehydration-induced proteins), intrinsically disordered proteins known to confer desiccation tolerance to plants (Murray and Graether, 2022). Dehydrins are part of the group 2 of the late embryogenesis abundant (LEA) family (Hernández-Sánchez et al., 2022). This family was first discovered in cottonseed embryos during the later stages of seed development, a period marked by natural dehydration (Dure et al., 1981). Expression of LEA genes has been shown to continue throughout the entire life cycle of the plant and in a wide range of tissue types, and are expressed ubiquitously throughout the plant kingdom (Hernández-Sánchez et al., 2022). All LEA proteins assist plants in the development of desiccation tolerance, but many specified roles have been identified for particular LEA proteins, such as DNA binding, protecting proteins from denaturation, and metal ion scavenging (Hernández-Sánchez et al., 2022).

By definition, a dehydrin must contain the lysine-rich, 15 residue K-segment. This segment may appear as many as 13 times (Close, 1996). A large database of dehydrin sequences was used to identify conserved features of this K-segment, which can be summarized by the following sequence: [XKXGXX(D/E)KIK(D/E)KXPG] (Malik et al., 2017). In addition to the many lysine residues, amino acid type is an important, conserved feature at certain positions within the segment. For example, hydrophobic amino acids are almost always found at positions five and six and negatively charged residues at positions seven and 11.

Dehydrins may contain several other conserved sequence motifs: the S-segment, Y-segment, and the F-segment (Close, 1996; Strimbeck, 2017). These conserved motifs are arranged in specific ways within the dehydrin sequence, and this is referred to as the sequence architecture, resulting the following arrangements: Y_n_SK_n_, Y_n_K_n_, K_n_S, SK_n_, FSK_n_, FK_n_, and K_n_, with the “n” indicating the number of times that a K-segment appears in the sequence (Close, 1996; Strimbeck, 2017; Riley et al., 2019). The Y-segment is defined by the presence of a central conserved tyrosine, although histidine or phenylalanine are also present at this position but at lower frequencies, indicating that the aromaticity of the residue is important. The consensus sequence of the Y-segment is DE(Y/H/F)GNPV (Malik et al., 2017). The F-segment is 12 residues long with the conserved sequence EXXDRG(L/M)FDFL(G/K) (Riley et al., 2019). The S-segment contains 4–8 contiguous serine residues, with conserved residues found before and after this tract. The consensus sequence has a 16-residue motif consisting of LHRSXSSSSSSSE(D/E)(E/D)(G/E/D), where the number of Ser residues varies (Malik et al., 2017). The S-segment is often tied to the K-segment by the conserved motif GXGGRRKK, prompting the suggestion that these two segments may play some functional role together (Malik et al., 2017). In between these conserved motifs are ϕ-segments, which are poorly conserved and highly variable in length. It has been suggested that the propensity towards small and polar amino acids results in the ϕ-segment being highly flexible (Malik et al., 2017), preventing the protein from forming secondary structure (Hughes and Graether, 2011). All together the highly hydrophilic and charged nature of dehydrin sequences causes them to be intrinsically disordered proteins, i.e. they contain little to no secondary or tertiary protein structure when alone in solution (Uversky, 2019).

The localization patterns of various dehydrins have provided insights into their potential protective functions. Dehydrins are most commonly found localized to the cytoplasm and nucleus, although some have been found in vacuoles, chloroplasts, mitochondria, and endoplasmic reticulum as well (Yu et al., 2018). Several studies have shown that S-segment can be phosphorylated, and that this modification may enhance nuclear localization. For example, the mutated wheat dehydrin WZY1-2, which lacked the S-segment, localized to both the nucleus and cytoplasm, while the wildtype was only found in the nucleus (Wang et al., 2020). The tomato dehydrin TAS14 is known to be phosphorylated *in vivo* (Torres-Schumann et al., 1991). Given that this protein contains several recognition sites for the casein kinase II (CK2), one study confirmed that TAS14 is phosphorylated by CK2 and cAMP-dependent protein kinase *in vitro* (Godoy et al., 1994). The maize dehydrin RAB17 was found to be in a mix of phosphorylated and unphosphorylated forms in both nuclear and cytosolic fractions following immunodetection and protein gel blot analysis (Goday et al., 1994). In the maize dehydrin DHN1, all seven serine residues of the S-segment are phosphorylated *in vivo*. When incubated with CK2 *in vitro*, phosphorylation levels increase over time to the level found *in vivo*, and that the phosphorylation sites corresponded to S-segment residues (Jiang and Wang, 2004). More recently, it was suggested that the kinase SnRK2.10 phosphorylates the S-segment of Arabidopsis dehydrins ERD10 and ERD14 (Maszkowska et al., 2019). *In vitro* phosphorylation experiments revealed that several kinases, including ABA-independent SnRK2 kinases, phosphorylated ERD10 and ERD14 under salt-stress. It was found through BiFC experiments that these dehydrins colocalize with SnRK2.10 in the cytoplasm *in vivo*. Finally, to test whether phosphorylation by SnRK2.10 affected localization patterns *in vivo*, the researchers created a pseudophosphorylated construct whereby SnRK2.10 target serine residues were substituted for glutamate to mimic phosphorylation (i.e. pseudophosphorylation). It was found that the Ser79Glu substitution facilitated nuclear transport, while the wild-type protein solely localized to the cytoplasm (Maszkowska et al., 2019).

While the effects on localization have been extensively examined, the effects of phosphorylation on dehydrin’s protective functions have not yet been established. We use the *Vitis riparia* dehydrin VrDHN1(a YSK_2_-type dehydrin) as a model for studying dehydrin structure and function. Multiple *in vitro* techniques were used to assess how phosphorylation may change protein cryoprotection, membrane association and DNA binding. We investigate whether pseudophosphorylation introduces some form of attenuation over dehydrin function, potentially shifting its protective roles in the nucleus.

## 2 Materials and methods

### 2.1 Construct design and cloning

Several pseudophosphorylated constructs were designed to assess the effect phosphorylation on the *in vitro* protective activity of the *Vitis riparia* dehydrin VrDHN1 (NCBI accession number XP_034683933). Three pseudophosphorylation (pp) constructs, which will be referred to as pp1VRDHN1, pp3VrDHN1, and pp5VrDHN1, contain an increasing number of glutamate substitutions within the S-segment (**Figure 1A**). The amino acid sequences were submitted to Twist Bioscience for the synthesis of genes optimized for protein expression in *E. coli*. For ligation into pET22b(+), NdeI and XhoI restriction sites were added to the 5’ and 3’ ends of the construct. Successful ligations were screened by restriction digest to check for the presence of an insert and subsequently by DNA sequencing.

**Figure 1:**
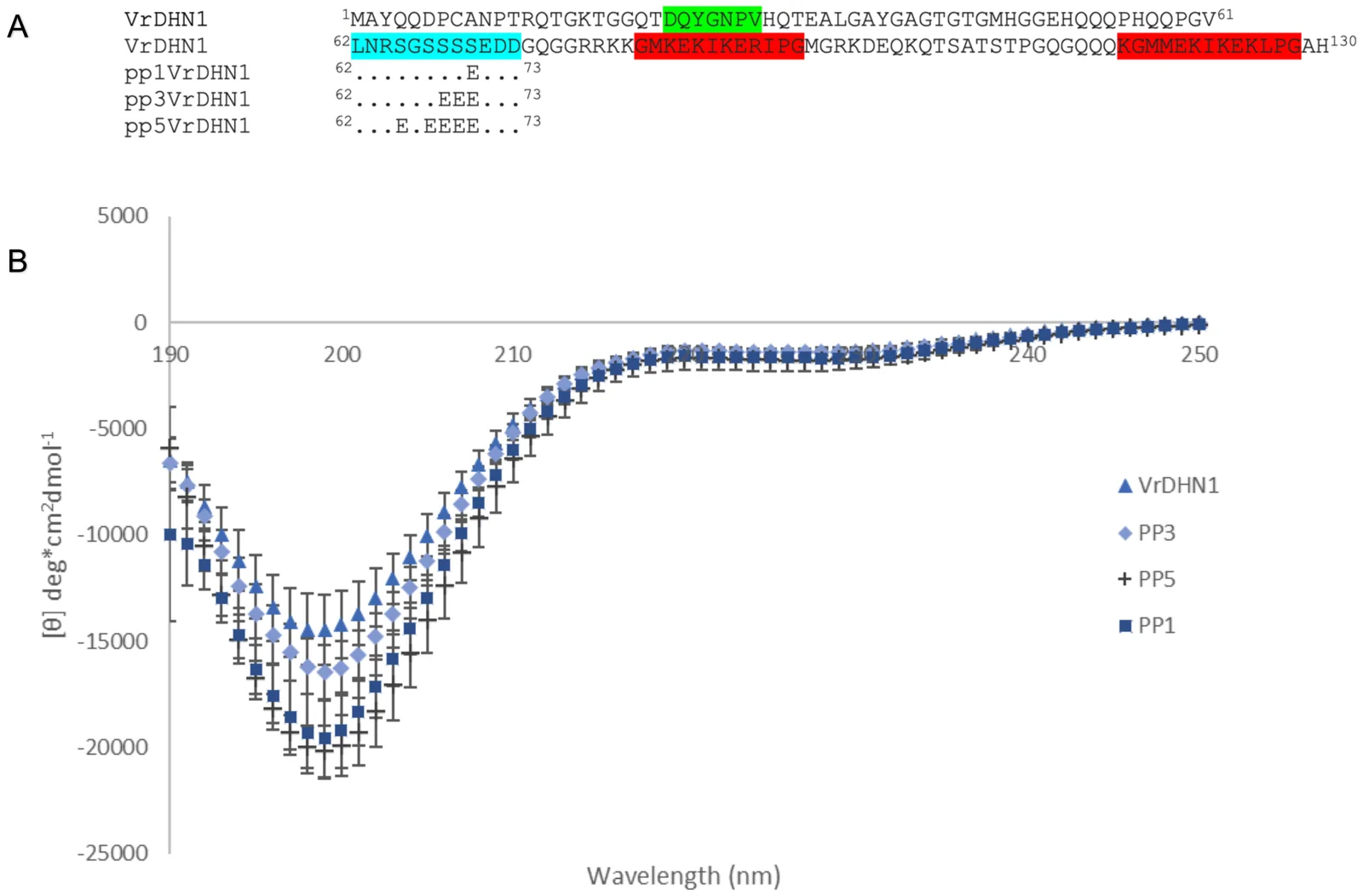
Protein sequence and structure of pseudophosphorylated VrDHN1 constructs. **(A)** Serine to glutamate substitutions were made to simulate phosphorylation of the S-segment. pp1VrDHN, pp3VrDHN and pp5VrDHN1 contain one, three and five substitutions, respectively. The alignment was made using ClustalW and the image was generated with Espript. Green – Y-segment, Cyan – S-segment, Red – K-segment. **(B)** Structural comparison of VrDHN1 and the pseudophosphorylated dehydrin constructs using circular dichroism. Constructs were measured in 10 mM Tris-HCl, pH 7.4 at 25°C from 190 to 250 nm. Each symbol represents the mean of n=3, with the error bars showing ± S.D.

### 2.2 Protein expression and purification

The *E. coli*-optimized constructs and wild-type VrDHN1 were purified as described by Hughes et al. (2013) with the exception that the protein was purified on a C4 column instead of a C18 column. The protein was then lyophilized overnight and stored at -20 °C until use. For the following assays, protein concentration was determined using a Pierce BCA protein assay kit.

### 2.3 LDH cryoprotection assay

The LDH assay was performed as described by Hughes and Graether (2011) with minor modifications. Briefly, LDH was dialyzed against 10 mM Na_2_HPO_4_, pH 7.4 and diluted to a final concentration of 50 µg/mL. Dehydrin constructs were dialyzed in 10 mM Na_2_HPO_4_ pH 7.4 and diluted to 5, 20, 50, 80, 120, 560, and 1000 µg/mL. LDH alone or in the presence of various protein additives was subjected to four cycles of freeze thaw treatments (30 s in liquid nitrogen followed by 5 min at 16 °C). Each reaction mixture contained 8 µL of LDH, 8 µL of protein additive, and 724 µL of reaction buffer (680.6 µL of 30 mM Na_2_HPO_4_, 21.7 µL of 2 mM NADH, and 21.7 µL of 10 mM pyruvic acid). The reaction buffer was added immediately prior to taking measurements. The conversion of NADH to NAD^+^ was monitored on a Cary UV/Vis Spectrophotometer for 2.5 minutes at 340 nm. Percent LDH activity was calculated by dividing the initial velocity of the consumption curve by the initial velocity of unfrozen LDH in the absence of protein additive (i.e. untreated LDH). Percent LDH activity was plotted as a function of protein additive concentration. To aid with data visualization, a trendline was added using the following formula, where x represents the protein additive concentration and x_0_ is the percent activity in the absence of protein. The variables a and b are fitted variables.

(1) $$\%LDH\;activity=\frac{a}{1+e^{-\frac{\left(x-x_{0}\right)}{b}}}$$

#### 2.3.1 SDS binding and circular dichroism

Dehydrin constructs (VrDHN1, pp1VrDHN1, pp3VrDHN1, and pp5VrDHN1), diluted to 10 μM, were dialyzed against 10 mM Tris-HCl pH 7.4 and subsequently filtered prior to data collection. Using a Jasco J-815 CD spectropolarimeter, six accumulations of each spectrum were collected from 190 to 250 nm, either alone or in the presence of 50 mM SDS. A bandwidth of 1 mm and quartz cuvette with a 1 mm pathlength were used for data collection. The spectrum of the Tris-HCl buffer was subtracted, and each spectrum was smoothed using a binomial smoothing algorithm. For the SDS binding experiments, the following equation was used to calculate α-helical structure, where the variable θ_222_ is the molar ellipticity at 222 nm (Morrow et al., 2000).

(2) $$\%\alpha -helicity=\frac{100*\left(3000-\theta_{222}\right)}{39000}$$

### 2.4 Electrophoretic mobility shift assay

The electrophoretic mobility shift assays (EMSAs) were performed as described by Boddington and Graether (2019). Briefly, 3 nM linearized pKTOL2 was incubated with EMSA buffer (5mM phosphate, 10mM NaCl, pH 7.4). Control samples had no protein added, while all other samples had 3, 6, or 9 μM of VrDHN1 or the pseudophosphorylated constructs. Agarose gels were run at 120 V for 5 min, and then at 80 V for 3 h.

### 2.5 Plasmid isoform assay

We performed the plasmid isoform assay to determine whether pseudophosphorylation affected the ability of VrDHN1 to protect from ROS damage. pETSUMO plasmid DNA encoding a gene for the NSs protein from La Crosse virus (Boddington and Graether, 2019) was isolated using a midiprep kit. The plasmid isoform assay was performed as described in Boddington and Graether (2019) and all steps were performed on ice. Briefly, all samples contained EMSA Buffer (5mM phosphate, 10mM NaCl, pH 7.4) and 4 nM pETSUMO La Crosse NSs plasmid. Samples contained either 10 μM of VrDHN1, ppVrDHN1, or control protein β-casein, while the positive (damage) control sample contained no protein. For the damaged samples, 6.67 mM H_2_O_2_ was added, and they were subsequently exposed to a Gel Doc trans UV illuminator for 5 min to induce hydroxy radical formation. To assess the level of damage, agarose gels were run at 120 V for 5 min, and then for 1 h at 100 V. The trace measurements (the area under the intensity profile curve in intensity x band width in mm) were taken for all bands and used to calculate the percentage of linear, supercoiled, and open circular DNA in each lane.

## 3 Results

### 3.1 Construct design

pp1VrDHN1 and pp3VrDHN1 pseudophosphorylated constructs were designed based on phosphorylation patterns identified by Goday et al. (1994) for CK2 sites, while pp5VrDHN1 was added to test the effect of increasing levels of phosphorylation (**Figure 1A**). To precisely control which residues were modified, we used pseudophosphorylation, where serine residues in the S-segment were replaced with glutamate to approximately mimic the size and charge of a phosphorylated serine residue. Circular dichroism (CD) was used to verify that the amino acid substitutions did not accidentally result in a gain in secondary structure. As can be seen in **Figure 1B**, the lack of change in the shapes of the CD spectra of the dehydrin constructs showed that the substitutions did not introduce any new secondary structure.

### 3.2 LDH assay

The protein cryoprotective function of dehydrins has been well-studied *in vitro*, with lactate dehydrogenase (LDH) being one of the most commonly used model enzymes (for example (Goyal et al., 2005; Hughes et al., 2013; Hara et al., 2017)), but the potential effects of phosphorylation, to our knowledge, have not been previously examined. The cryoprotection assays performed here were compared using the four dehydrin constructs, BSA (positive control) and lysozyme (negative control). All pseudophosphorylated constructs were able to effectively protect LDH from damage (**Figure 2**). BSA, a known cryoprotectant, was also able to protect LDH, but differences were observed in terms of maximum recovery. All constructs were able to achieve >100% activity recovery (i.e. the dehydrins were able to recover more activity after freeze-thaw treatment than the untreated LDH in the absence of any protein). Specifically, VrDHN1 restored 138% activity, meaning that LDH was functioning at a higher catalytic activity in the presence of these proteins than the baseline control (unfrozen LDH without protein cryoprotectant). pp3VrDHN1 and pp5VrDHN1 restored 139% and 145% LDH activity respectively. pp1VrDHN1 restored approximately 125% LDH activity, which is slightly less than the other dehydrin constructs. However, VrDHN1 and all pseudophosphorylated dehydrin constructs restored a higher %LDH activity than the positive control BSA (which was ~100%). The lysozyme control shows that the protection is not a generic effect of any protein, since only maximally 30% activity was recovered.

**Figure 2:**
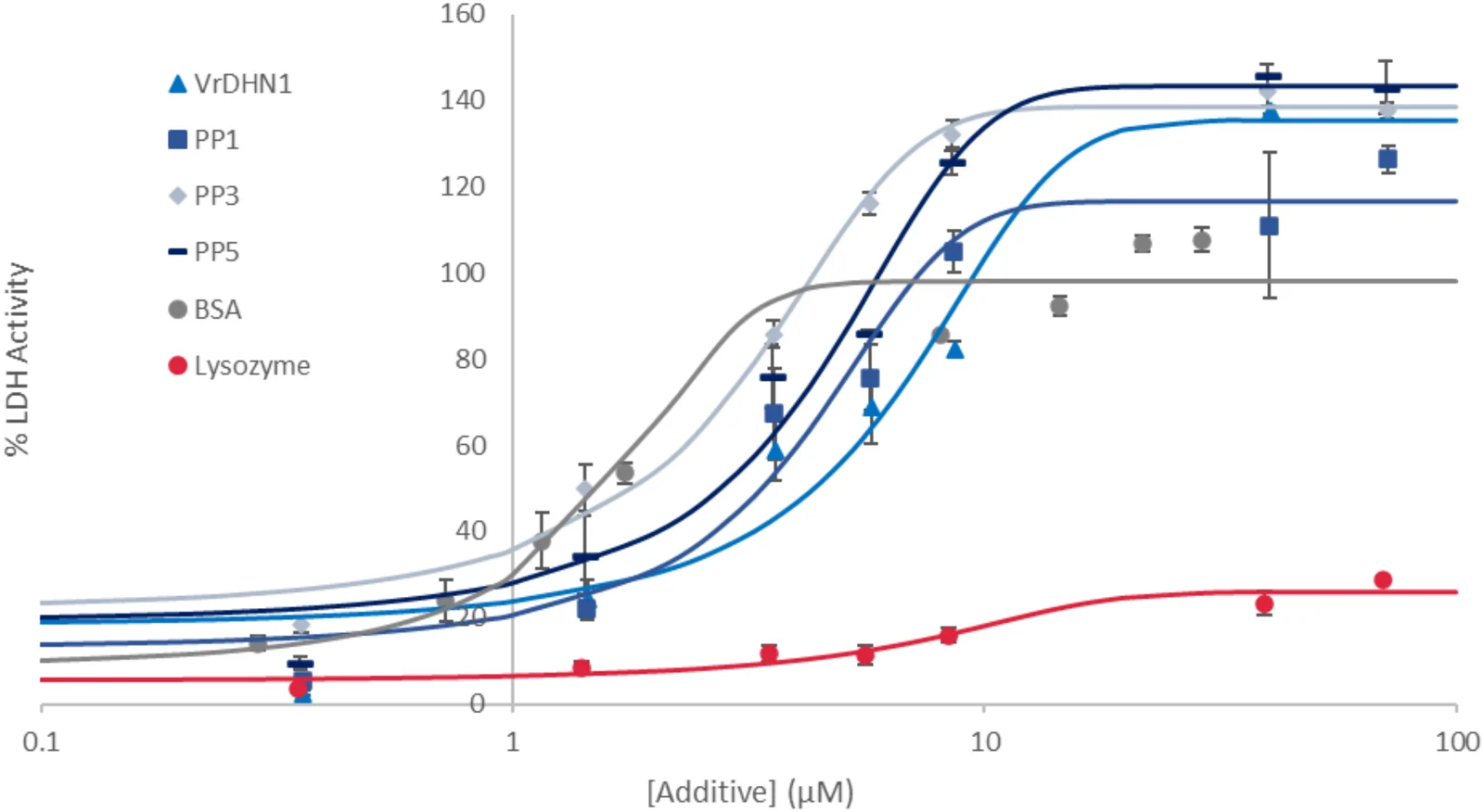
Cryoprotection of LDH activity by pseudophosphorylated dehydrin constructs. LDH was subjected to freeze-thaw treatment alone and in the presence of protein additives. Percent LDH activity is calculated based on the activity of unfrozen LDH without protein additive. The line represents the fit using Equation 1, with *x* being the additive concentration. Dehydrin constructs: n=5; lysozyme and BSA: n=3. Error bars represent the ± S.D.

Another way to quantify cryoprotection is to use PD_50_ values, i.e. the protective dose required to restore 50% LDH activity, where smaller values show increased efficiency. We found that the PD_50_ was slightly reduced in the pseudophosphorylated constructs when compared to the wildtype (**Table 1**). Among the dehydrin constructs pp3VrDHN1 had the lowest PD_50_ followed by pp5VrDHN1, pp1VrDHN1, and VrDHN1 (**Table 1**), though the PD_50_ shift was statistically significant between the wild-type protein and pp3VrDHN1 and pp5VrDHN1.

**Table 1:** LDH cryoprotective assay results with VrDHN1 and pseudophosphorylated constructs.

Construct	%LDH activity restored	PD_50_ (μM)	SD	p-value
VrDHN1	138	4.4	0.41	
pp1VrDHN1	126	3.4	0.64	0.061
pp3VrDHN1	139	1.8	0.12	0.003
pp5VrDHN1	145	2.8	0.14	0.005

The PD_50_ and percent LDH activity restored were determined in the LDH cryoprotection assay. PD_50_ was calculated using Equation 1. Student’s t-test was used to determine statistical significance of PD_50_ values in comparison to wild-type VrDHN1, with a p-value cutoff of 0.05. .

### 3.3 SDS binding

Dehydrins have been shown to reduce membrane damage *in vivo* (reviewed in Murray and Graether, 2022). Membrane binding assays are a valuable tool in understanding how dehydrins can interact with membranes. Because dehydrins have been shown to preferentially interact with negatively charged membranes, we examined whether the introduction of negative charges through pseudophosphorylation could affect binding.

CD experiments were subsequently used to measure the gain in α-helicity of the dehydrin constructs in the presence of SDS micelles. The ellipticity of the constructs was measured from 190 to 250 nm in the presence and absence of 50 mM SDS (**Figure 3**). Each of the constructs showed that SDS caused a gain in molar ellipticity at 200 nm, and a reduction at 222 nm, indicative of a gain in α-helicity and loss of disorder. Interestingly the pseudophosphorylated constructs gained a greater percent α-helicity than VrDHN1 in the presence of SDS, with the gain exhibited by pp5VrDHN1 being statistically significant. An SDS titration was performed to show that the increase in α-helicity pp5VrDHN1 could be observed over a range of SDS concentrations (**Figure 4**; **Table 2**).

**Figure 3:**
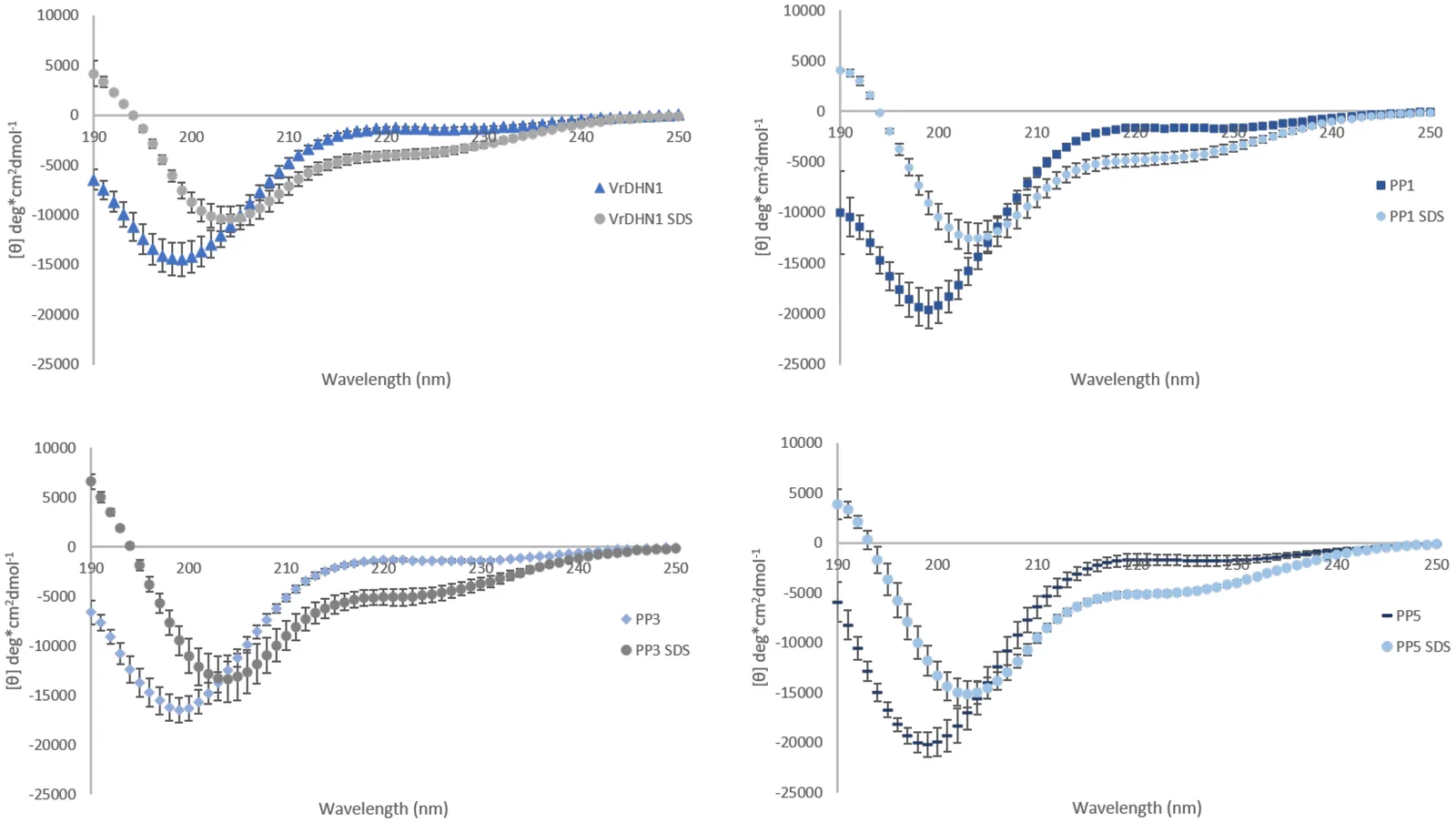
CD Spectra of VrDHN1 and pseudophosphorylated constructs in the presence and absence of SDS. Molar ellipticity of pseudophosphorylated dehydrin constructs in the presence of 50 mM SDS, a membrane mimetic (n=3, mean ± S.D.). Dehydrin and constructs were diluted to 10 μM and measured in 10 mM Tris-HCl, pH 7.4 at 25 °C.

**Figure 4:**
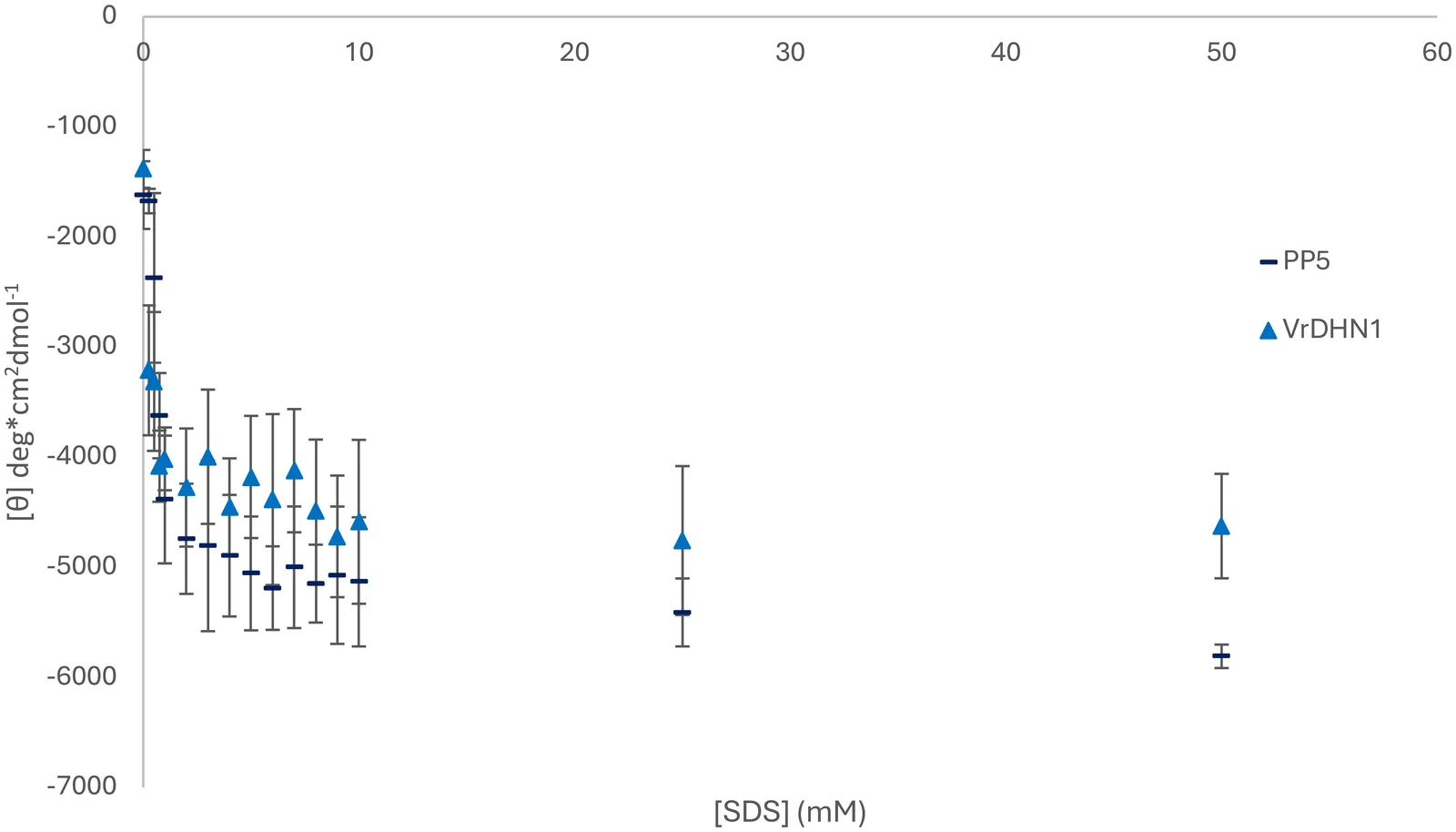
Change in α-helicity of pp5VrDHN1 in the presence of SDS. Molar ellipticity at 222 nm (absolute value) of VrDHN1 and pseudophosphorylated constructs alone and in the presence of a range of SDS concentrations (absolute value; n=3, mean ± S.D.). Error bars represent standard deviation. The above data were obtained from CD spectra containing 10 μM dehydrin constructs and measured in 10 mM Tris-HCl, pH 7.4 from 190 to 250 nm at 25 °C.

**Table 2:** SDS binding assay results with VrDHN1 and pseudophosphorylated constructs.

Construct	% gain in α-helicity	SD	p-value
VrDHN1	6.7	1.2	
pp1VrDHN1	7.7	1.5	0.375
pp3VrDHN1	9.6	2.1	0.086
pp5VrDHN1	8.7	1.3	0.038

The percent gain in α-helicity was determined using circular dichroism in the presence of 50 mM SDS. Constructs were measured in 10 mM Tris-HCl, pH 7.4 at 25 °C from 190 to 250 nm. Percent α-helicity was determined using Equation 2. Student’s t-test was used to determine statistical significance relative to wild-type VrDHN1 with a p-value cutoff of 0.05. .

### 3.4 Nucleic acid binding and protection

Since many dehydrins localize to the nucleus, it has long been proposed that they may be protecting DNA from ROS damage through binding. VrDHN1 has been shown to bind DNA in a sequence independent manner (Boddington and Graether, 2019). This property was explored using the electrophoretic mobility shift assay (EMSA), where the binding of the protein to a linearized plasmid causes its mobility to be slowed. The assumption is that the negatively charged phosphates in the nucleic acid backbone interact with the multiple lysine residues, which have a positive charge. Again, the question was asked whether the presence of the negatively charged pseudophosphorylated residues could interfere with binding. We therefore performed the mobility shift assays using pseudophosphorylated VrDHN1 constructs (**Figure 5**). For the wild-type VrDHN1, the plasmid is shown to shift, and the extent of shift increases with increasing protein concentration as presumably more protein binds (**Figure 5**; lanes 3-5). The shifts in the presence of the pseudophosphorylated constructs were reduced with the level of modification, with the pp5VrDHN1 construct resulting in no observable shift (**Figure 5**; lanes 6-8).

**Figure 5:**
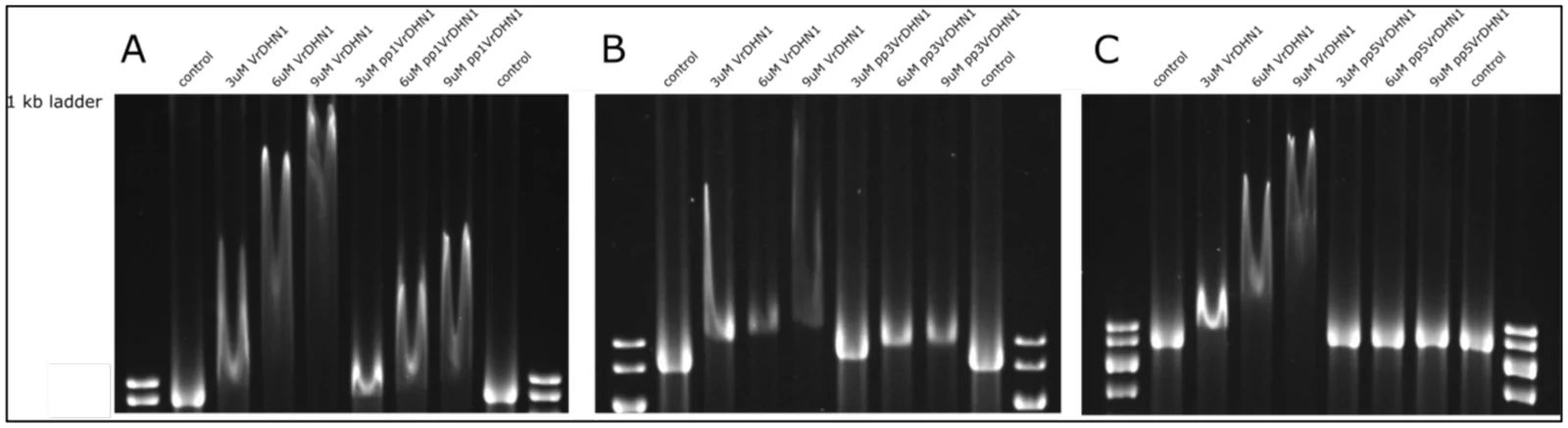
Binding of VrDHN1 and pseudophosphorylated constructs to DNA. Electrophoretic mobility shift assay of 3 nM pkTOL2 plasmid DNA in 5 mM phosphate, 10 mM NaCl, pH 7.4. Lanes 3–5 show pkTOL2 in the presence of 3, 6, and 9 µM VrDHN1 and lanes 6–8 show pkTOL2 in the presence of 3, 6, and 9 µM of various pseudophosphorylation constructs of VrDHN1: **(A)** pp1VrDHN1 **(B)** pp3VrDHN1 **(C)** pp5VrDHN1. Flanking 1 kb DNA ladder, as well as flanking controls of pkTOL2 alone, are also shown in their respective lanes.

Previous studies have shown that dehydrins are able to protect nucleic acids from ROS damage (Hara et al., 2003; Boddington and Graether, 2019). The next experiment was to determine whether this reduction in binding resulted in a lowered ability to protect DNA from ROS damage. We performed the plasmid isoform assay where the percentage of supercoiled, open circular, and linear DNA was determined for plasmid DNA with added H_2_O_2_ and UV light exposure to induce ROS damage (**Figure 6**). Surprisingly, the amount of supercoiled DNA recovered was not significantly different between VrDHN1 and all three of the pseudophosphorylated constructs (**Table 3**), whereas the β-casein control was unable to recover any supercoiled DNA, suggesting that the increased negative charge did not adversely affect protection.

**Figure 6:**
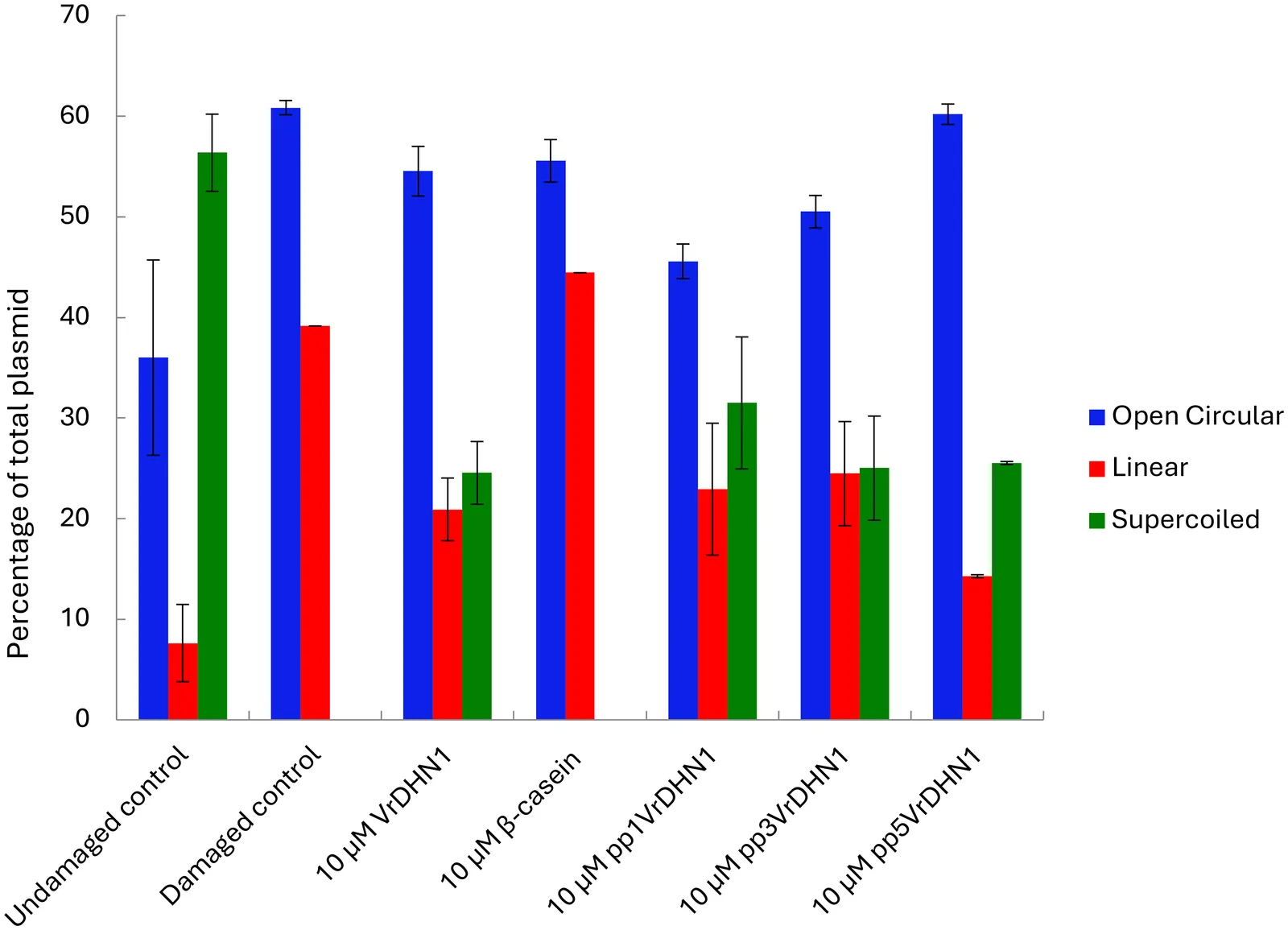
Protection of DNA from H_2_O_2_-induced damage. Plasmid isoform assay of 4 nm pETSUMO-La Crosse NSs plasmid DNA in the presence of pseudophosphorylated constructs. The undamaged control did not contain H_2_O_2_, and the damaged control received 6.67 mM H_2_O_2_ exposed to UV light to induce hydroxyl radical formation. VrDHN1, pp1VrDHN1, pp3VrDHN1, pp5VrDHN1, and β-casein samples were done in triplicate and contained 10 µM protein. Standard error bars are shown.

**Table 3:** Plasmid isoform assay results with VrDHN1 and pseudophosphorylated constructs.

Construct	Percent supercoiled plasmid	SD	p-value
VrDHN1	24.4	3.0	
pp1VrDHN1	31.3	6.6	0.1746
pp3VrDHN1	24.9	5.3	0.8938
pp5VrDHN1	25.4	0.2	0.5954

The amount of supercoiled plasmid DNA was measured using densitometry. Student’s t-test was used to determine statistical significance relative to wild-type VrDHN1 with a p-value cutoff of 0.05.

## 4 Discussion

While phosphorylation has long been known to be play a role in dehydrin nuclear localization, recent work has begun to address its role in plant protection. The expression of several kinases, such as *Arabidopsis* SnRK2, has been shown to be associated with the abiotic stress tolerance response (Maszkowska et al., 2021). The expression of AsSnRK2D from oat caused improved salt and drought tolerance in tobacco, as well as an upregulation of several dehydrin genes (Xiang et al., 2020). The expression of HbDHN3 dehydrin from *Hordeum brevisubulatum* improved salt stress in *Arabidopsis*, and this effect was further enhanced by its phosphorylation (Feng et al., 2025). In the same study, a pseudophosphorylation mutant of HbDHN3 with modifications at putative HbCK2 sites exhibited improved growth during salt stress compared to the version that could not be phosphorylated. The connection between certain kinases and dehydrins may be twofold: through increased expression levels and phosphorylation-induced changes in localization and function. Here, we use a pseudophosphorylation approach in order to control the precise location of modified amino acids and examine whether the modification introduces some form of fine-tuning over dehydrin function in the three major protective roles: protein cryoprotection, membrane binding and DNA binding.

### 4.1 Pseudophosphorylation improved the PD_50_ of VrDHN1 in LDH cryoprotection

A major and well-studied *in vitro* function of dehydrins is their ability to maintain protein structure and function after a freeze-thaw stress, where protection likely works by preventing aggregation (Hughes et al., 2013; Drira et al., 2015; Szlachtowska and Rurek, 2023). We assessed whether pseudophosphorylated constructs are able to protect LDH from freeze-thaw induced damage as effectively as the wild-type dehydrin. As seen in **Table 1**, the pp3VrDHN1 and pp5VrDHN1 constructs had statistically significant lower PD_50_ values compared to VrDHN1, i.e. less protein was required to restore 50% LDH activity. Additionally, pp3VrDHN1 and pp5VrDHN1 restored a slightly higher percent LDH activity compared to VrDHN1, while the maximal percent activity restored by pp1VrDHN1 was slightly reduced (**Figure 2**; **Table 1**). The improved LDH cryoprotection by the pseudophosphorylated constructs could be due to the added negative charge of the protein, resulting in a more extended conformation of the dehydrin S-segment, enhancing the molecular shield effect. It has been speculated that the positively charged K-segments contribute to the interaction between the dehydrin and LDH through long-range electrostatics without direct binding (Hughes et al., 2013); it is possible that this cluster of negative charge functions in a similar way, attracting the dehydrin to positively charged regions on the surface of LDH resulting in a more even distribution around LDH, and therefore more effectively preventing aggregation. This does not necessarily mean that more phosphorylation will result in even more efficient protection, since it was found that too many negative charges, such as in the disordered FROST protein, resulted in poor protection of LDH (Newman et al., 2017).

### 4.2 Pseudophosphorylation resulted in an increase in α-helicity in the presence of SDS

We tested whether pseudophosphorylation affects membrane binding *in vitro*, since the plasma membrane is one of the first sites of damage due to abiotic stress. SDS has been shown to induce α-helical character in interacting proteins and contains negatively charged sulfates, which mimic the negatively charged phosphate head groups found in phospholipids. In this study, SDS binding assays were performed to determine whether the addition of pseudophosphoryl groups (i.e. negative charge) affected the ability of VrDHN1 to gain α-helicity in the presence of membranes and hence binding. Surprisingly, pp1VrDHN1, pp3VrDHN1, and pp5VrDHN1 gained a higher percent α-helicity than VrDHN1 (**Figure 3**). pp5VrDHN1 was the only construct to show a statistically significant gain in α-helicity and this increase was observed over a range of SDS concentrations (**Figure 4**). A study on K-segments suggested that the Asp/Glu flanking the Lys residues in the K-segment were important for orienting the Lys residues towards the micelle surface and stabilizing the helix (Clarke et al., 2015); a similar effect may be happening with the pseudophosphorylated constructs. In this way the pseudophosphorylated residues would be pointed away from the membrane surface and therefore not able to interfere with binding.

### 4.3 Nucleic acid protection

We lastly examined the potential effect of phosphorylation on nucleotide binding and protection from ROS damage. In this case the negatively charged, pseudophosphorylated residues had an effect, where their presence reduced DNA binding, with five pseudophosphorylated residues abolishing it (**Figure 5**). While the phosphorylation state of VrDHN1 has not yet been examined, dehydrins such as RAB17, ERD10, and ERD14 have been shown to be phosphorylated between three and six times *in vivo* (Goday et al., 1994; Maszkowska et al., 2019), suggesting that the pp5VrDHN1 is a plausible maximum. The elimination of the gel shift that was seen in pp5VrDHN1 is logical considering the large negative charge that pseudophosphorylation adds to the protein. Since the association of VrDHN1 to DNA has been proposed to be an electrostatic association between the positive charges on VrDHN1 and the negatively charged phosphate backbone of DNA (Boddington and Graether, 2019), the addition of five negatively charged groups was expected to interfere with this interaction.

Despite the reduction in gel shift, there was no significant difference seen in the amount of recovered supercoiled DNA when pp1VrDHN1, pp3VrDHN1, and pp5VrDHN1 were added to the plasmid isoform assay (**Figure 6**). At first glance this may suggest that DNA binding is not required for its protection from ROS damage, but this does not appear to be the case. A possibility is that the pp5VrDHN1 protein is still binding to the DNA, but with an affinity below the detectable limit of the assay. Support from this comes from the results using β-casein, another disordered protein. β-Casein is a protein which cannot bind DNA (Boddington and Graether, 2019), yet it is unable to recover any supercoiled DNA in the plasmid isoform assay (**Figure 6**). If pp5VrDHN1 were entirely unbound, it would be expected to result in a similar level of protection as β-casein. Furthermore, the protection conferred to DNA by pp5VrDHN1 implies that the binding strength of the interaction between VrDHN1 and DNA need only be minimal for protection to occur, and that phosphorylation of the S-segment, should it be required for nuclear localization, would not interfere with the ability of VrDHN1 to protect DNA from oxidative damage.

## 5 Conclusion

Here, we investigated whether pseudophosphorylation of VrDHN1 affected its protective roles *in vitro* to gain insight on how phosphorylated dehydrins may function *in vivo*. The effects of pseudophosphorylation on protein, membrane and DNA protection were different, showing that phosphorylation did not always result in a change in function despite the addition of a potentially deleterious negative charge. Pseudophosphorylation tended to increase the ability of VrDHN1 to protect LDH from freeze-thaw damage and also increased its ability to bind to the negatively charged SDS micelles. With regards to the LDH assay, this result could be because the hydrodynamic radius of the protein is increased due to electrostatic repulsion within the dehydrin. The added glutamate residues may orient the interacting lysine residues towards the membrane surface, which may explain why the gain in structure in the presence of SDS was increased. The EMSA and plasmid isoform assays were used to assess DNA binding and protection by pseudophosphorylated VrDHN1. While the added negative charge hindered the DNA binding of VrDHN1 as seen in the EMSA, it had no effect on the protection from ROS damage compared to the wild-type VrDHN1. Overall, the data shows that phosphorylation may enhance the ability for dehydrins to protect certain proteins and lipids from abiotic stress, while having little effect on DNA protection from ROS damage. Whether phosphorylation has a tuning effect on dehydrin function can be further studied by determining phosphorylation state in different cellular compartments *in vivo* under different stress conditions. Further work is required to determine the sites of *in vivo* phosphorylation and if these also result in improvements in protective function.

## Data Availability

The raw data supporting the conclusions of this article will be made available by the authors, without undue reservation.
